# In silico investigations identified Butyl Xanalterate to competently target CK2α (CSNK2A1) for therapy of chronic lymphocytic leukemia

**DOI:** 10.1038/s41598-022-21546-0

**Published:** 2022-10-21

**Authors:** Suliman A. Alsagaby, Danish Iqbal, Iqrar Ahmad, Harun Patel, Shabir Ahmad Mir, Yahya Awaji Madkhali, Atif Abdulwahab A. Oyouni, Yousef M. Hawsawi, Fahad A. Alhumaydhi, Bader Alshehri, Wael Alturaiki, Bader Alanazi, Manzoor Ahmad Mir, Waleed Al Abdulmonem

**Affiliations:** 1 Department of Medical Laboratory Sciences, College of Applied Medical Sciences, Majmaah University, AL-Majmaah, 11952 Kingdom of Saudi Arabia; 2 Division of Computer Aided Drug Design, Department of Pharmaceutical Chemistry, R. C. Patel Institute of Pharmaceutical Education and Research, Shirpur, Maharashtra 425405 India; 3 Department of Biology, Faculty of Sciences, University of Tabuk, Tabuk, Kingdom of Saudi Arabia; 4 Genome and Biotechnology Unit, Faculty of Sciences, University of Tabuk, Tabuk, Kingdom of Saudi Arabia; 5 Research Center, King Faisal Specialist Hospital and Research Center, P.O. Box 40047, Jeddah, 21499 Kingdom of Saudi Arabia; 6 College of Medicine, Al-Faisal University, P.O. Box 50927, Riyadh, 11533 Kingdom of Saudi Arabia; 7 Department of Medical Laboratories, College of Applied Medical Sciences, Qassim University, Buraydah, Kingdom of Saudi Arabia; 8 Biomedical Research Administration, Research Center, King Fahad Medical City, Riyadh, Kingdom of Saudi Arabia; 9 Prince Mohammed bin Abdulaziz Medical City, AlJouf, Kingdom of Saudi Arabia; 10 Department of Bioresources, School of Biological Sciences, University of Kashmir, Srinagar, India; 11 Department of Pathology, College of Medicine, Qassim University, Qassim, Kingdom of Saudi Arabia

**Keywords:** Biochemistry, Biotechnology, Cancer, Computational biology and bioinformatics, Molecular biology, Biomarkers, Molecular medicine, Oncology

## Abstract

Chronic lymphocytic leukemia (CLL) is an incurable malignancy of B-cells. In this study, bioinformatics analyses were conducted to identify possible pathogenic roles of CK2α, which is a protein encoded by *CSNK2A1*, in the progression and aggressiveness of CLL. Furthermore, various computational tools were used to search for a competent inhibitor of CK2α from fungal metabolites that could be proposed for CLL therapy. In CLL patients, high-expression of *CSNK2A1* was associated with early need for therapy (n = 130, *p* < 0.0001) and short overall survival (OS; n = 107, *p* = 0.005). Consistently, bioinformatics analyses showed *CSNK2A1* to associate with/play roles in CLL proliferation and survival-dependent pathways. Furthermore, PPI network analysis identified interaction partners of CK2α (PPI enrichment *p* value = 1 × 10^–16^) that associated with early need for therapy (n = 130, *p* < 0.003) and have been known to heavily impact on the progression of CLL. These findings constructed a rational for targeting CK2α for CLL therapy. Consequently, computational analyses reported 35 fungal metabolites out of 5820 (filtered from 19,967 metabolites) to have lower binding energy (ΔG: − 10.9 to − 11.7 kcal/mol) and better binding affinity (Kd: 9.77 × 10^7^ M^−1^ to 3.77 × 10^8^ M^−1^) compared with the native ligand (ΔG: − 10.8, Kd: 8.3 × 10^7^ M−^−1^). Furthermore, molecular dynamics simulation study established that Butyl Xanalterate-CK2α complex continuously remained stable throughout the simulation time (100 ns). Moreover, Butyl Xanalterate interacted with most of the catalytic residues, where complex was stabilized by more than 65% hydrogen bond interactions, and a significant hydrophobic interaction with residue Phe113. Here, high-expression of *CSNK2A1* was implicated in the progression and poor prognosis of CLL, making it a potential therapeutic target in the disease. Butyl Xanalterate showed stable and strong interactions with CK2α, thus we propose it as a competitive inhibitor of CK2α for CLL therapy.

## Introduction

Chronic lymphocytic leukemia (CLL) is a B-cell neoplasm and is the commonest adult leukaemia in Western countries with reported incidence rates in the USA and Europe being 4–6 per 100,000/ annually^1,2^ The clinical outcomes of CLL are extremely heterogeneous, where overall survival (OS) and time-to-first treatment (TTFT) vary drastically across CLL patients^3^. Early need for therapy (short TTFT) and short OS are characteristics of progressive and aggressive form of CLL, whereas late or no need for therapy and long OS are features of indolent form of the disease. Several prognostic markers have been reported to predict the clinical course of the disease. For instance, the mutational status of immunoglobulin genes (*IGHV*); mutated *IGHV* is associated with good prognosis, whereas unmutated *IGVH* predicts poor prognosis. In addition, chromosomal aberrations, such as deletions in 17p and 11q are characteristics of high-risk CLL^4^. Furthermore, increased protein expression of CD38, CD49d, ZAP-70 and CXCR4 is associated with an aggressive form of CLL^3,5–7^.

Despite the great effort and research that have been made in the area of cancer therapy^8–15^, CLL remains incurable and life threatening especially for those with poor prognosis^16,17^. Casein kinase II subunit alpha (CK2α; a protein encoded by *CSNK2A1*) is a catalytic subunit of a constitutively active serine/threonine-protein kinase complex that phosphorylates a wide range of substrates and regulates a diverse of cellular processes, such as cell proliferation, apoptosis, haematopoiesis, resistance to cytotoxin agents, protein stability and chaperon activities^18^. In malignancies including CLL, CK2α was reported to be over-expressed^19,20^. CK2α enhances cellular viability and proliferation through PI3K/AKT, Wnt/β-catenin and JAK/STAT dependent signaling mechanisms^21,22^. Therefore, targeting CK2α was shown to induced apoptosis in CLL cells^23–25^. Similar findings were also reported in CLL cells xenografted in mice^26^. Interestingly, targeting the expression of CK2α in CLL cells isolated from patients with poor prognosis and chemotherapy resistance due to chromosomal alternations (11q and 17p deletions) induced apoptosis^27^. Collectively, these findings argue that CLL patients may benefit from therapeutic strategies of targeting CK2α.

Several inhibitors of CK2α have been reported^28^, one of which is CX-4945 (Silmitasertib) that has been designated as an orphan drug by FDA for cancer treatment^29,30^. Nevertheless, CX-4945 has some limitations, such as restricted selectivity because it exerts inhibitory effect on twelve other kinases and shows a stronger binding affinity with Clk2 (one of the twelve kinases) compared to CK2α^31^. Moreover, therapeutic resistance to CX-4945 due to various factors like drug uptake, drug efflux, gene mutations, pathway alteration and target inactivation are of great concern^32,33^.

Natural products and their derived compounds are believed to be a rich source of therapeutics that could be employed in the treatment and management of several communicable and non-communicable diseases^34–48^. Natural products like metabolites from fungus and plants have been considered to be safe and economical with great bioactive potentials against multidrug-resistant cancers^49,50^. Fungi were reported to have variety of metabolites which proves to be blockbuster drugs, such as Caspofungin, Cyclosporine, Finglomide, Lovastatin and many more. Nearly 40% of new chemical entities approved by the United State Food and Drugs Administration (US FDA) are of natural origin and most of them are fungal metabolites. Hence, the metabolites originated from fungi have great potential and prominent role in therapeutic drug discovery^51–53^.

Although several pieces of evidence have shown the value of targeting CK2α for CLL therapy, information about how CK2α contributes to the disease progression and worse clinical outcomes of CLL remains scarce in the literature. Therefore, in current work, we first studied the impact of *CSNK2A1* expression on OS and TTFT of CLL patients and conducted bioinformatic investigations^54,55^ to identify possible roles of *CSNK2A1* in CLL progression. Consequently, this constructed a rational for targeting CK2α in CLL. In silico approach-based search for kinase competent inhibitors have been reported to be effective^56–61^, Therefore, we used various computational tools to search for a competent inhibitor of CK2α from fungal metabolites that could be proposed for CLL therapy.

## Methodology

### Transcriptomics data sets

Transcriptomics data sets of CLL available in GEO (accession number: GSE22762^62^ and GSE39671^63^ were used to study the impact of CK2α transcript expression on the prognosis and progression of CLL. These two data sets were selected for four reasons. First, the transcriptomics analysis was conducted on CLL cells isolated from peripheral blood of CLL patients. Second, the two data sets included prognostic information of CLL patients on whose samples the transcriptomics analysis was conducted. The data set (GSE22762) included OS data and the data set (GSE39671) contained TFTT data. To the best of our knowledge these two data sets are the only CLL transcriptomics data with OS and TTFT information available in GEO. Third, the two data sets were generated from two separate CLL cohorts with > 100 patients each (GSE22762 = 107 patients; GSE39671 = 130 patients). Fourth, the same oligonucleotide microarray platform (Affymetrix Human Genome U133 Plus 2.0 Array) was used to produce the two transcriptomics data sets. This was an important inclusion criterion because it reduces the possible variation that could rise if the two data sets had been produced using different platforms of oligonucleotide microarray. The files (type: DataSet SOFT) of the transcriptomics data sets were downloaded from GEO and used.

### Functional profiling

Functional profiling of the genes that correlated with *CSNK2A1* (PS =  > 0.60) was performed using the gProfiler (https://biit.cs.ut.ee/gprofiler/gost)^64^. The analysis was conducted against four known databases: Gene Ontology (GO) database (http://geneontology.org/)^65,66^, KEGG pathway database (https://www.genome.jp/kegg/)^67^, Reactome pathways database (https://reactome.org/)^68^ and WikiPathways database (https://www.wikipathways.org/index.php/WikiPathways)^69^. The option “only annotated genes” was selected for statistical domain scope and corrected *p* value cut-off was set at ≤ 0.05. The calculation of corrected p value was conducted on the basis of Benjamini–Hochberg method.

### Protein–protein interaction network analysis

Protein–protein interaction (PPI) analysis and network construction were conducted using the “Search Tool for the Retrieval of Interacting Genes” (STRING; https://string-db.org/)^70^. The following criteria were applied: homo sapiens was chosen for organism; full STRING network was selected for the network type; confidence was chosen for the meaning of network edges; all active sources for interaction were selected. Only PPIs with enrichment score < 0.001 were reported. Next, file generated from STRING was loaded into Cytoscope (version 3.4.0; https://cytoscape.org/)^71^ for network visualization.

### Prediction of physicochemical, medicinal chemistry, and ADME-T properties

Total of 19,967 compounds from fungus database of PubChem (accessed on: 04/12/2021) was filtered out to get drug like metabolite on the basis of their physiochemical properties, medicinal chemistry parameters and blood–brain barrier permeability through SwissADME (http://www.swissadme.ch) web-based tool^72^. Furthermore, the ADMET analysis was performed for 10 best hits (best docking score in comparison with a standard reference) of fungal metabolites such as, absorption, and metabolism was predicted through SwissADME whereas distribution and excretion were predicted through Admetlab2.0^73^ web-based tool^74^. Moreover, the toxicity potential and LD50 was predicted through ProTox-II^75^ web-based tool^76^.

### Preparation of ligands and protein

The fungal metabolites (filtered compounds of fungus database) were used as ligand to check their inhibitory potential through computational study^77^. The “.sdf” file of ligands (3D conformers) were retrieved from fungal database of PubChem (https://pubchem.ncbi.nlm.nih.gov/). These ligands were then energy minimized using universal force field (UFF), further the PyRx-python 0.8 software (https://sourceforge.net/projects/pyrx/) inbuilt tool (OpenBabel) was used to convert them into Autodock suitable (file format “.pdbqt”). Furthermore, we extracted the high resolution (1.60 Å) 3D co-crystallized structure of human protein kinase CK2α subunit (PDB Id: 3PE1) with native ligand (CX-4945) from online freely available protein data bank (PDB) database (accessed on: 25th November 2021 (http://www.rcsb.org/pdb/)^78,79^. The protein was converted into Autodock suitable format after removing all heteroatoms, water molecule and adding polar hydrogens.

### Molecular docking

Computational screening of fungal metabolites against the target protein (CK2α; PDB Id: 3PE1) was performed via PyRx-python 0.8 software (https://sourceforge.net/projects/pyrx/) based on Autodock 4.2 tool^46,80,81^. The interactions was analysed with the help of Discovery Studio Visualizer ((*BIOVIA Discovery Studio - BIOVIA—Dassault Systèmes*®, 2021)^82^. The grid box dimensions was set to 25 × 25 × 25 Å, and centered at 22.77 × − 29.95 × 14.46 Å^83^.

### Molecular dynamic simulation study

The conformational flexibility, stability, and binding interaction of the promising compounds Butyl Xanalterate and Fumiquinazoline Q docked conformation into CK2α (PDB Id: 3PE1) in dynamic conditions were examined using MD simulation. MD simulations were conducted in the Desmond module of the Schrödinger 2020-1 suite, which has been installed on an Ubuntu 18.04 (HP Z2 G2 TOWER) workstation (with an NVIDIA Quadro 6000 4 GB graphics processing unit (GPU)) system. Autodock generated ligand–protein complexes are imported into the Schrodinger's Maestro interface. The "*protein preparation wizard*" was employed to refine the ligand–protein complex structures by adjusting formal charges, assigning bond orders, and correcting side and backbone chains^84,85^. Upon properly accomplishing the *protein preparation wizard* module, ligand–protein complexes solvated using simple point charge (SPC) water molecules, which was defined as an orthorhombic box with a minimum distance of 10 Å from the protein surface to the box's sides. To make each system electrically neutral, counter ions (Na^+^ and Cl^−^) were added through the system builder module, and salt (NaCl) at a concentration of 0.15 M was provided to simulate physiological conditions^86,87^. Using a hybrid algorithm of the steepest descent and the limited-memory Broyden–Fletcher–Goldfarb–Shanno (LBFGS) algorithms in the OPLS3e force field, the solvated system was treated for energy minimization to eliminate stearic collisions among protein and solvated water molecules^88,89^. In NPT ensembles (isothermal-isobaric) with 100 ps intervals between trajectory snapshots, the simulation was run for 100 ns. Temperature of 300 K and a pressure of 1 bar during simulation is maintain through the Nose–Hoover chain thermostat and Martyna-Tobias-Klein barostat controllers, respectively^90,91^.

### Post simulation binding free energy analysis

The molecular mechanics combined with Generalized Born surface area (MM/GBSA) approach was used to calculate the post-simulation binding free energies (**ΔG**_Bind_) of ligand–protein complexes. The binding free energy (**ΔG**_Bind_) based on MM/GBSA was calculated using the *thermal_mmgbsa.py* script. The binding free energy was computed using a 0–1000 ns MD simulation trajectory with the VSGB solvation model associated with the OPLS3e force field with 10-step sampling size (every ns) as input for the MM/GBSA analysis. The Prime MM/GBSA binding free energy (kcal/mol) is evaluated using the law of additivity, which combines different energy modules such as hydrogen bonding, van der Waals, columbic, lipophilic, covalent, solvation, π- π stacking’s, and self-contact of ligand and protein were combined collectively^92^.

### Statistical analyses

Kaplan–Meier curves were constructed using Prism Graphpad software (version 7; https://www.graphpad.com/guides/prism/7/user-guide/index.htm) and *p* values with hazard ratios (HRs) were calculated using the Log-rank test. Correlation analysis and Pearson score calculations were performed using Excel software (version 14.4.0). The *p* values and the FDRs of the functional profiling analysis were calculated using the gProfiler^64^. The *p* value of PPI enrichment analysis was calculated using STRING (https://string-db.org/)^70^. Cluster analysis using average linkage method for clustering and Manhattan method for distance measurement was conducted using Heatmapper web-based tool (http://www.heatmapper.ca/)^93^. Heatmapper was also employed to construct heatmaps.

## Results and discussion

### Implication of *CSNK2A1* in the progression and prognosis of CLL

**Figure 1:**
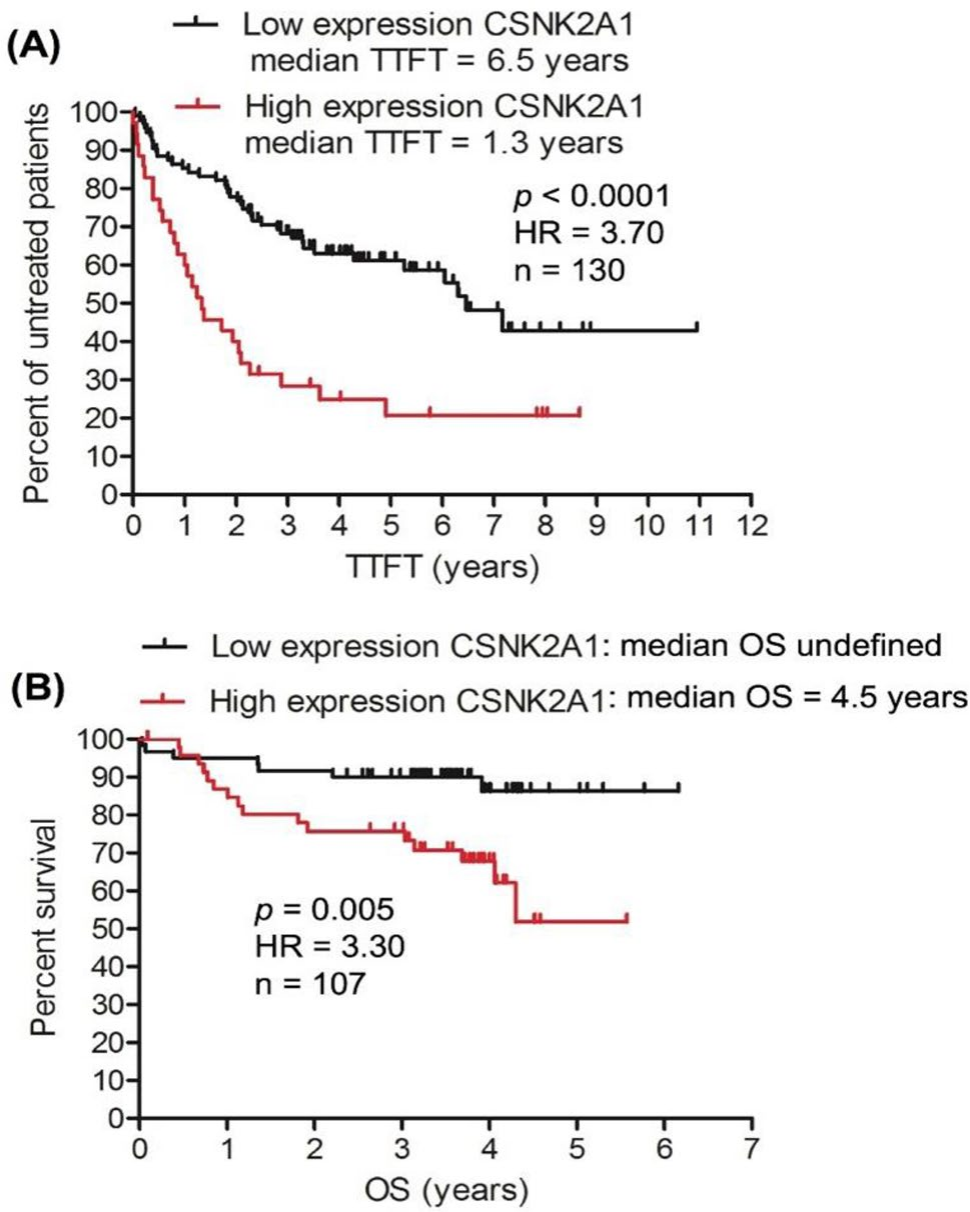
High expression of *CSNK2A1* gene is associated with short TTFT and short OS in CLL patients. Two CLL transcriptomics data sets from GEO were used for the analysis; TTFT analysis was done on the data set GSE39671 (**A**) and the OS analysis was performed on the data set GSE22762 (**B**). GEO: gene expressing omnibus; HR: hazard ration of high-expression versus low-expression.

OS and TTFT are very important clinical measures of CLL prognosis^94^. In contrast to indolent form (good prognosis) of CLL, the progressive and aggressive form of the disease (poor prognosis) is characterized by short TTFT and short OS^3^. Investigation was conducted to determine whether the expression of *CSNK2A1* in CLL cells is associated with short TTFT and short OS of CLL patients. The analysis was performed on two CLL transcriptomics data sets from GEO (accession number: GSE22762^62^ and GSE39671^63^. As shown by Kaplan–Meier curve (Fig. 1A), increased expression of *CSNK2A1* is associated with short TTFT in CLL patients; the median TTFT in the high-expression group was 1.3 years compared with 6.5 years in the low-expression group (n = 130, *p* < 0.0001, HR of high-expression versus low-expression = 3.70). Likewise, Kaplan–Meier curve also showed that high-expression of *CSNK2A1* was associated with short OS; the median OS was 4.5 years for the high-expression group and was undefined for the low-expression group (Fig. 1B, n = 107, *p* = 0.005, HR of high-expression versus low-expression = 3.30). These findings provided evidence for the implication of *CSNK2A1* in CLL progression and poor clinical outcomes, supporting previous studies that involved *CSNK2A1* in the survival and proliferation of CLL cells^21–23,27,95,96^. In line with our findings, earlier studies also showed an association between increased expression of *CSNK2A1* and poor prognosis of other malignancies, such as acute myeloid leukaemia^97^, hepatocellular carcinoma^98^, ovarian cancer^99^ and colorectal cancer^100^. Nevertheless, to the best of our knowledge, this is the first work to point to the poor prognostication (as indicated by short TTFT and short OS) of CLL by increased expression of *CSNK2A1*.

### Functional insight into the pathogenic role of *CSNK2A1* in CLL

**Figure 2:**
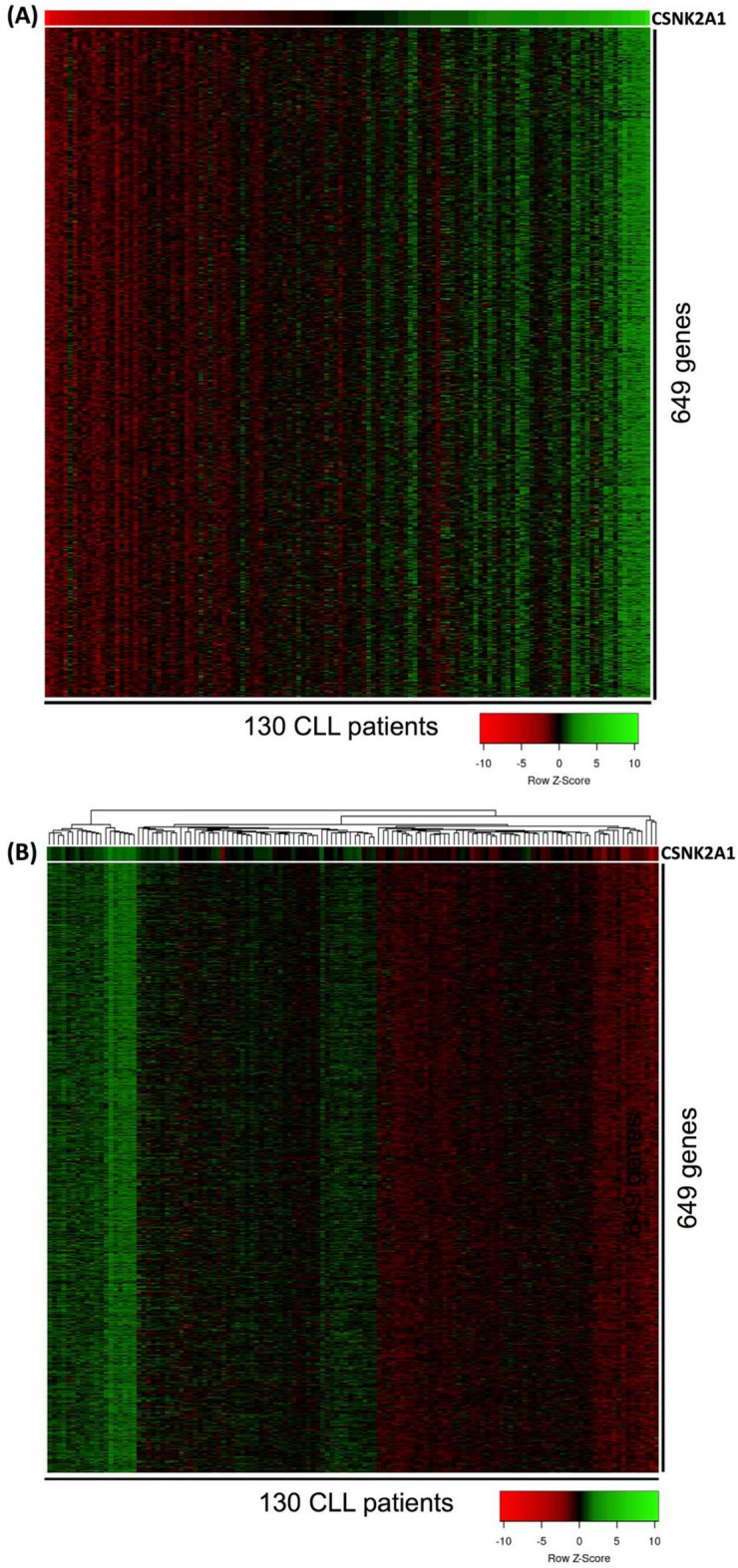
Heatmap presentation of the correlation between the expression of *CSNK2A1* and the expression of other genes from the entire transcriptome of CLL cells in 130 patients. In (**A**) the 130 patients were sorted according to the expression of *CSNK2A1* (top gene) from least expression (red color) to the highest expression (green color). From the whole transcriptome of CLL cells, a total of 649 genes were found to have their expression correlated with the expression of *CSNK2A1* in the 130 patients (Pearson score = 0.60–0.81; *p* value < 0.00001). Cluster analysis using average linkage method for clustering and Manhattan method for distance measurement clustered the 130 patients according the expression of *CSNK2A1* and the 649 genes (**B**). The CLL transcriptomics data set used in this analysis was GSE39671. Color legend: red is low expression and green is high expression.

Although several studies showed CK2α to play roles in the survival and proliferation of CLL cells^23,27,95,96^, the pathogenic roles of CK2α in CLL remains not fully understood. Therefore, here, attempts were made to explain the association of *CSNK2A1* expression with the progressive and aggressive form of CLL (short TTFT and OS). Consequently, correlation analysis using Pearson score (PS) was conducted between the expression of *CSNK2A1* and the transcriptome expression of CLL cells from 130 patients (data set: GSE39671). As a result, 649 genes were found to have their expression correlated significantly with the expression of *CSNK2A1* in the 130 patients (PS = 0.60–0.81; *p* value < 0.00001; Fig. 2A; Supporting data 1). In addition, the 130 patients were found to cluster according to the expression *CSNK2A1* and the expression of the 649 genes (Fig. 2B). Interestingly, genes that have been known to play roles in the progression and poor prognosis of CLL, such as the anti-apoptotic gene *API5*^75^, *DEK* oncogene^101^, *SET* oncogene^102^, transcriptional factor *STAT1*^103^ and *HMGB1*^104^ had expression that correlated with the expression of *CSNK2A1* (Supporting data 1) in CLL cells from the 130 patients.

**Figure 3:**
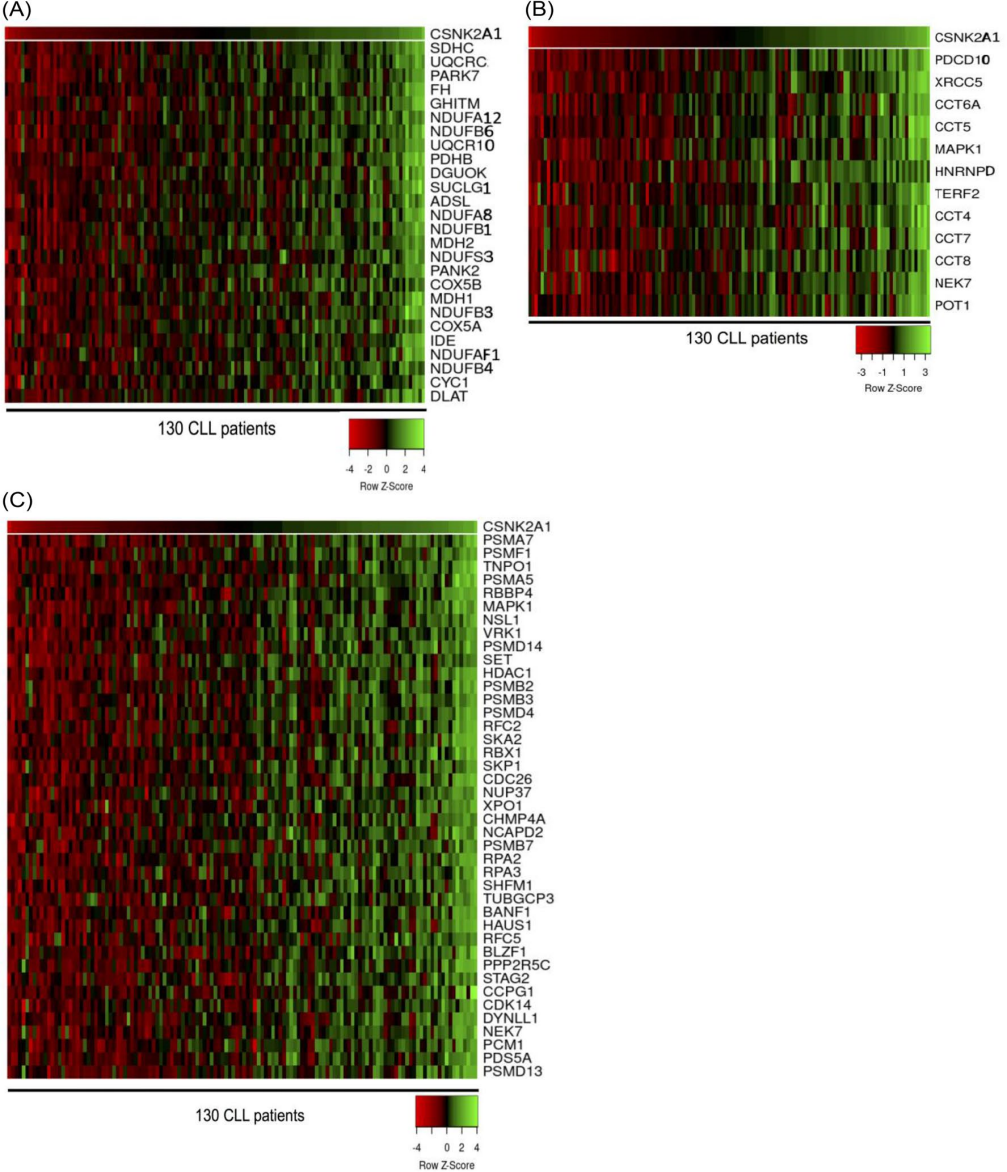
Heatmap presentation of the correlation between the expression of *CSNK2A1* and the expression of genes that enriched CLL-related pathways. The expression *CSNK2A1* was shown at the top of each heatmap; and patients (n = 130) were sorted from the least expression (red color) to the highest expression (green color) of *CSNK2A1*. Heatmap (**A**) shows the correlation between the expression *CSNK2A1* and the expression of aerobic respiration genes (Pearson score = 0.77–0.60; *p* value < 0.00001). Heatmap (**B**) demonstrates the correlation between the expression *CSNK2A1* and the expression of genes that positively regulate telomere maintenance (Pearson score = 0.81–0.60; *p* value < 0.00001). Heatmap (**C**) presents the correlation between the expression *CSNK2A1* and the expression of cell cycle mitotic genes (Pearson score = 0.71–0.60; *p* value < 0.00001). The correlation analysis was conducted on the CLL transcriptomics data set (GSE39671). Color scale: red is low expression; green is high expression.

Next, functional profiling of the 649 genes was performed using gProfiler against four databases (GO database, KEGG pathway database, Reactome pathways database and WikiPathways database). Table 1 shows the significantly enriched pathways (corrected *p* value < 0.05) by the 649 genes. Heatmap presentation of the correlation between the expression of *CSNK2A1* and the expression of genes that enriched for aerobic respiration, telomere maintenance and cell cycle mitotic in the 130 CLL patients are shown in Fig. 3. Interestingly and consistent with the association of increased expression of *CSNK2A1* with the progression and poor clinical outcomes of CLL, the enriched pathways, such as mRNA splicing, aerobic respiration, oxidative phosphorylation, production of ATP, telomere maintenance, B-cell receptor signaling, nuclear factor-kappaB (NF-kB) signaling, DNA synthesis and cell cycle, have been shown to heavily contribute to the progression and poor prognosis of CLL^105–110^. In fact, CLL cells were found to rely greatly on the fore mentioned pathways for their proliferation and survival. As a result, targeting many of these pathways has been shown to induce apoptosis in CLL cells. Furthermore, our results agree with earlier in vitro reports, where in cells other than CLL cells, *CSNK2A1* was implicated mRNA splicing^111^, BCR signalling^112^ and nuclear factor-kappaB signaling^113^. Taken these findings together, it is possible that *CSNK2A1* mediates its pathogenic impact on CLL through its association with/roles in the pathways that favour the progression of CLL; such as BCR, NF-kB, telomere maintenance, mRNA splicing and aerobic respiration.

**Table 1:** Functional profiling of 649 genes that correlated with *CSNK2A1* in CLL patients.

Term ID	Pathway term	Corr *p* value	Term size	Intersection size
GO:0000398	mRNA splicing, via spliceosome	1.3 × 10^−6^	360	37
GO:0009060	Aerobic respiration	3.8 × 10^−6^	200	26
REAC:R-HSA-5389840	Mitochondrial translation elongation	1 × 10^−5^	87	17
REAC:R-HSA-5368286	Mitochondrial translation initiation	1 × 10^5^	87	17
REAC:R-HSA-5419276	Mitochondrial translation termination	1 × 10^5^	87	17
GO:0042775	Mitochondrial ATP synthesis coupled electron transport	4 × 10^5^	99	17
REAC:R-HSA-1234174	Cellular response to hypoxia	5 × 10^5^	75	15
REAC:R-HSA-72172	mRNA Splicing	0.0003	186	23
REAC:R-HSA-69239	Synthesis of DNA	0.0003	120	18
REAC:R-HSA-1428517	The citric acid (TCA) cycle and respiratory electron transport	0.0004	175	22
REAC:R-HSA-1234176	Oxygen-dependent proline hydroxylation of Hypoxia-inducible Factor Alpha	0.0004	66	13
REAC:R-HSA-5676590	NIK noncanonical NF-kB signaling	0.0008	59	12
KEGG:00020	Citrate cycle (TCA cycle)	0.0009	30	8
REAC:R-HSA-5607761	Dectin-1 mediated noncanonical NF-kB signaling	0.001	60	12
GO:0032206	Positive regulation of telomere maintenance	0.001	52	11
GO:0032212	Positive regulation of telomere maintenance via telomerase	0.002	34	9
REAC:R-HSA-5687128	ERK3/ERK4 signaling	0.002	89	14
REAC:R-HSA-1168372	Downstream signaling events of B Cell Receptor (BCR)	0.004	80	13
REAC:R-HSA-69278	Cell Cycle, Mitotic	0.01	549	41
REAC:R-HSA-1169091	Activation of NF-kappaB in B cells	0.02	67	11
GO:0006119	Oxidative phosphorylation	0.02	151	17
GO:2000573	Positive regulation of DNA biosynthetic process	0.02	67	11
GO:0042254	Ribosome biogenesis	0.03	335	27
GO:0006260	DNA replication	0.04	338	27

Functional profiling analysis was conducted for the genes (n = 649) that correlated with *CSNK2A1* in CLL patients (n = 130) using gProfiler (https://biit.cs.ut.ee/gprofiler/gost) against gene ontology database (http://geneontology.org/)^65,66^, KEGG pathway database (https://www.genome.jp/kegg/)^67^, Reactome pathways database (https://reactome.org/)^68^ and WikiPathways database (https://www.wikipathways.org/index.php/WikiPathways)^69^. Corr *p* value, corrected *p* value that was calculated on the basis of Benjamini–Hochberg false discovery rate (FDR). Term size, number of all genes that have been known to function in a pathway term. Intersection size, number of genes (from those that correlated with *CSNK2A1*) that were assigned to a pathway term.

### Protein–protein interaction network analysis

**Figure 4:**
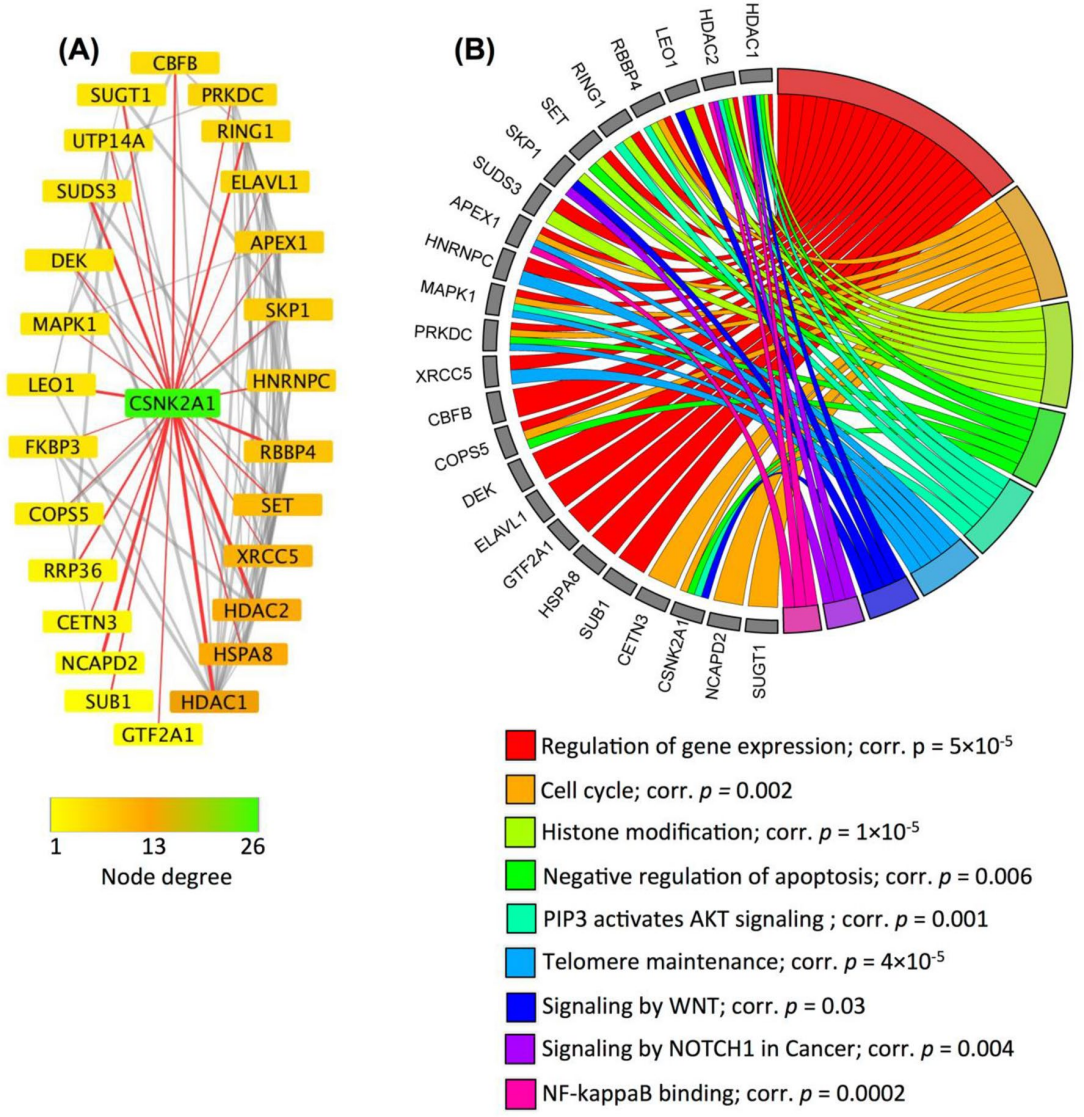
Protein–protein interaction network of CK2α (a protein encoded by *CSNK2A1*). STRING and Cytoscape were used for PPI network analysis of the protein products corresponding to the 649 genes to identify binding partners of CK2α (*CSNK2A1*). The analysis identified 26 interaction partners of CK2α (**A**). Functional profiling using gProfiler was conducted for the CK2α interaction partners and was visualized in chord plot (**B**). All terms shown in the chord plot were significantly enriched with corrected *p* value (corr. *p*) < 0.05.

**Figure 5:**
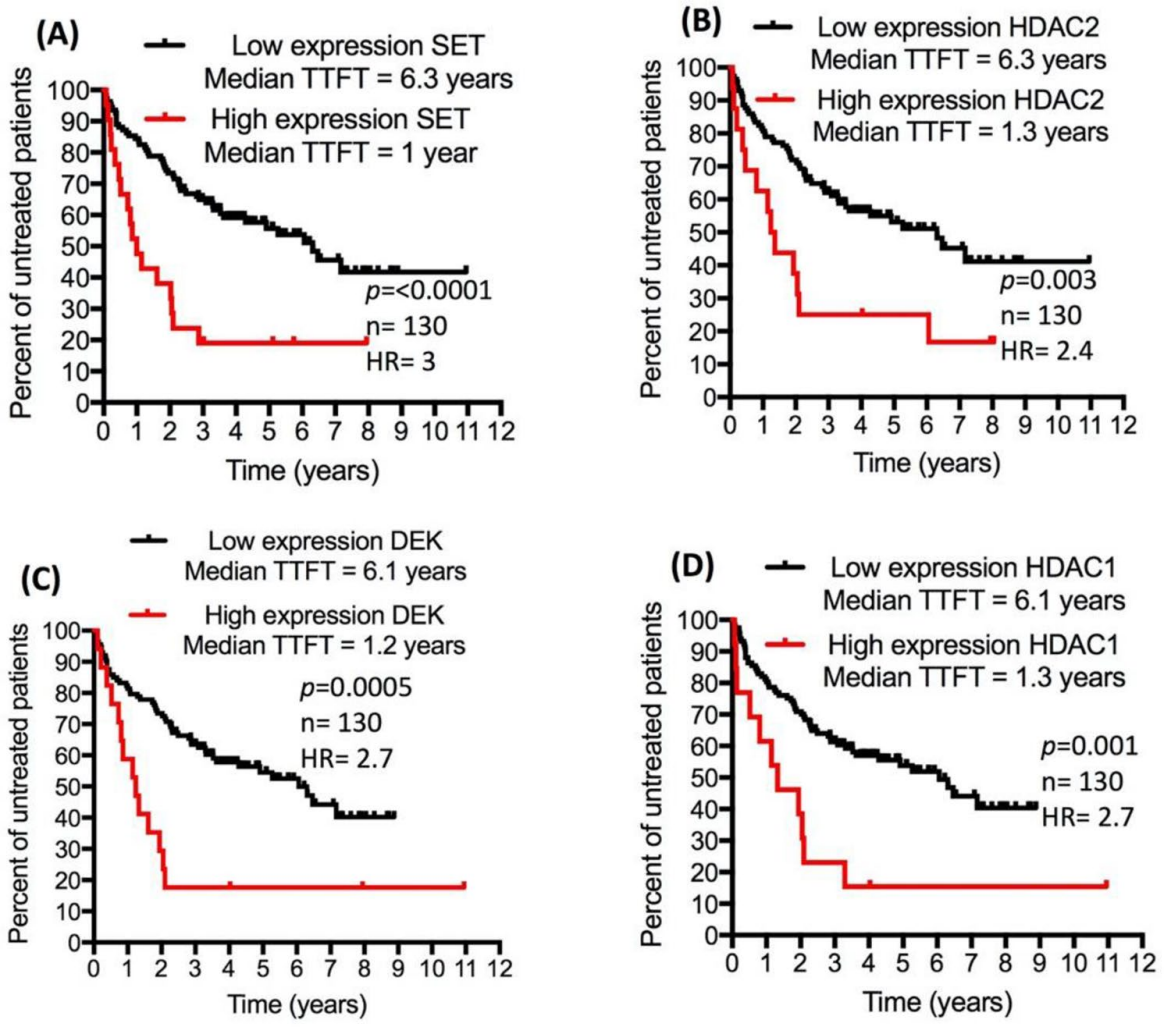
Increased gene expression of CK2α interaction partners is associated with short TTFT. CLL transcriptomics data set GSE39671 was used to investigate if the transcript expression of CK2α phosphorylation targets is associated with early need for therapy (which is a reflection of a progressive and poor prognosis CLL). High gene expression of SET (**A**), HDAC2 (**B**), DEK (**C**) and HDAC1 (**D**) was associated with short TTFT. HR, hazard ration of high-expression versus low-expression; TTFT, time-to-first-treatment.

To determine whether interactors of CK2α exist among the protein product of the 649 genes, PPI network analysis was conducted using STRING and Cytoscape. Figure 4 shows the PPI network, where proteins are represented by nodes, interaction between proteins is represented by edges; and node degree denotes number of binding partners of a protein. The analysis identified 26 nodes, 76 edges (as opposed to 22 edges expected) with average node degree of 5.85 and PPI enrichment *p* value = 1 × 10^−16^. Top node degrees were found for CK2α (node degree = 26), HDAC1 (node degree = 13) and HSPA8 (node degree = 10). Next, we investigated whether CK2α targeted proteins for phosphorylation like SET^114^, DEK^115^, HDAC1^116^ and HDAC2^116^ possessed transcript expression that associate with the aggressiveness of CLL. As shown by Kaplan–Meier curves (Fig. 5), increased gene expression of SET, DEK HDAC1 and HDAC2 was found to significantly associate with the need for early treatment (short TTFT; n = 130, *p* ≤ 0.003). As mentioned earlier, short TTFT is a characteristic of a progressive and aggressive form of CLL. Our findings support previous reports that identified binding partners of CK2α included SET oncoprotein, DEC oncoprotein, PRKDC, RBBP4, HDAC1 and HDAC2 to play important roles in the progression and poor prognosis of CLL^101,102,117–119^. Overall, these findings may provide further insights into the pathogenic roles of CK2α in CLL.

The findings reported above; (1) the association of increased expression of *CSNK2A1* with short TTFT and short OS, (2) the correlation between the expression of genes implicated in CLL survival and proliferation-dependent pathways with the expression of *CSNK2A* in CLL cells, (3) identification of CK2α interaction partners that associate with the aggressiveness of CLL; support each other and establish a rational for targeting CK2α for CLL therapy. Therefore, in the subsequent sections of the study, different computational tools were utilized to search for competent inhibitors of CK2α that could be proposed for CLL therapy.

### Physicochemical properties of compounds

In this study we have filtered out the whole fungus database of 19,967 metabolites reported in PubChem by applying sequentially the specific range of these drug-likeness criteria and Lipinski rule of 5, such as molecular weight (100 to 500 g/mol), rotatable bond (0 to 9), hydrogen bond donor (0 to 5), hydrogen bond acceptor (0 to10), XLogP (− 1 to 5), polar surface area (20–140 Å^2^). After applying these filters, we funneled down 12,589 metabolites. Furthermore, we applied few other filters by SWISSADME and funneled down 5820 metabolites who showed high GI (gastrointestinal) absorption, molar refractivity (40–140), Fcsp3 higher than 0.25, and blood brain barrier negative (Table 2).

**Table 2:** Physiochemical and medicinal chemistry properties of top 10 best hits.

Code	PubChem CID	Compound name	Formula	Physicochemical property								Medicinal chemistry	
				MW	HA	RB	HBA	HBD	MR	TPSA	XLOGP3	Lipinski #violations	Fraction Csp3
C1	139590691	Butyl Xanalterate	C24H22O7	422.43	31	5	7	3	114.41	113.29	4.45	0	0.33
C2	139586689	Ergosecaline	C24H28N4O4	436.5	32	4	5	3	127.56	103.53	2.76	0	0.46
C3	44581698	15-Chlorotajixanthone hydrate	C25H27ClO6	458.93	32	4	6	3	126.43	100.13	4.79	0	0.4
C4	10429091	Paeciloquinone E	C20H16O7	368.34	27	0	7	3	93.4	113.29	2.84	0	0.3
C5	44255150	See below^a^	C26H28O7	452.5	33	4	7	2	125.33	101.66	3.93	0	0.42
C6	44567617	Penicitrinol A	C23H26O5	382.45	28	0	5	2	107.26	68.15	4.46	0	0.48
C7	114895	Altertoxin III	C20H12O6	348.31	26	0	6	2	86.67	99.66	2.43	0	0.3
C8	139584842	Fumiquinazoline Q	C23H21N5O4	431.44	32	2	6	3	126.43	116.56	0.16	0	0.3
C9	146683113	(+)-8-Hydroxysclerodin	C18H16O7	344.32	25	0	7	3	92.13	117.2	1.87	0	0.33
C10	44255148	Ruguloxanthone A	C25H26O6	422.47	31	4	6	2	120.37	96.97	4.56	0	0.36
NL	24748573	Silmitasertib	C19H12ClN3O2	349.8	25	3	5	2	98.56	75.1	4.4	0	0

^a^Compound name of CID “44255150: is “(1R,2S)-8-[(R)-[(2S)-3,3-dimethyloxiran-2-yl]-methoxymethyl]-1,11-dihydroxy-5-methyl-2-prop-1-en-2-yl-2,3-dihydro-1H-pyrano[3,2-a]xanthen-12-one”.

The importance of these parameters to be followed was well established and stated that most of the drugs failed during drug development process because their inefficiency to follow these criteria^120,121^. The physiological parameters like molecular weight (MW), hydrogen bond donors (HBD), hydrogen bond acceptor (HBA), and XlogP of majority of orally active medications were found to be in specific range (MW: 160–500 g/mol, HBD: ≤ 5, HBA: ≤ 10, Xlog P: − 1 to ≤ 5). More than 10 rotatable bonds of chemical structure represent the poor oral bioavailability^122^. For better intestinal absorption the molar refractivity (MR) range was considered between 40 and 130. Therefore Lipinski et al., defines that for the drug-likeness the compounds must follow the acceptable range of at least three properties out of five physiochemical properties^120^.

Moreover, absorption, distribution, metabolism, excretion, and toxicity (ADMET) analysis have been shown in Table 3. We have finalized the fungal metabolites who showed high GI absorption and negative for BBB permeability. For the absorption analysis we selected two parameters such as GI absorption and Pgp-Substrate. As stated, before that all the metabolites exhibited high GI absorption, but five metabolites (C2-C6) found to be PGP-substrate. To evaluate the distribution, we opted two parameters namely plasma protein bound (PPB) percentage (%) and blood brain barrier permeability. We have already selected only those metabolites who showed negative BBB permeability means they cannot cross the BBB. PPB (%) is well recognize parameter for distribution and those compounds exhibited more than 90% of PPB value do not have good distribution^74^. Our results showed that four fungal metabolites (C1, C3, C4, C6) including reference compound (Native ligand: NL) have higher than 90% value for PPB. The proper metabolism of drugs is important step for the better medicinal activity and lower toxicity where metabolism may get effected due to the inhibition of cytochromes. Those compounds do not show inhibitory effect on cytochromes (CYP) are consider having good metabolism^123^. Our results illustrate that C6 and C8 do not inhibit all the considered CYPs (1A2, 2C19, 2C9, 2D6, and 3A4), C2 inhibits one CYP-3A4, C3, C5, and C7 inhibits two cytochromes, C1, C4, C9, C10 inhibits three cytochromes and reference compound have inhibitory effect on four cytochromes. We checked the two criteria to understand the excretion of metabolites namely CL, and T1/2^74^. The results stated that all the best hits have good excretion as their half-life of a drug (T1/2) value is less than 0.7 but reference compound showed T1/2 value nearly equivalent to 0.7. High value for fifty percent concentration of lethal dose (LD50) are good for medicinal drugs. Our results showed that C6 have highest LD50 (4738 mg/kg) among all the best hits followed by C4 (3000 mg/kg), C9 (1600 mg/kg), C1 (1190 mg/kg), C8 (1100 mg/kg), and rest showed lower than 1000 mg/kg of LD50. From our results of toxicity, we noticed that except C1 and C8 fungal metabolite all the other metabolites have carcinogenic and/or mutagenic activity. C1 showed hepatotoxicity and immunogenic activity, C8 did not showed any toxicity. Whereas NL (reference compound) exhibited hepatotoxicity activity.

**Table 3:** ADME-T profile of top 10 best hits.

Code	Absorption		Distribution		Metabolism (Inhibitors of CYP)					Excretion		Toxicity					
	GIA	Pgp-S	PPB (%)	BBB + ve	CYP-1A2	CYP-2C19	CYP-2C9	CYP-2D6	CYP-3A4	CL	T1/2	Pred. LD50	Dilli	Carcino	Immuno	Mutagen	Cyto
C1	High	No	97.1	No	No	No	Yes	Yes	Yes	5.924	0.276	1190 mg/kg	Active (0.69)	Inactive (0.62)	Active (0.96)	Inactive (0.97)	Inactive (0.93)
C2	High	Yes	65.7	No	No	No	No	No	Yes	10.061	0.475	300 mg/kg	Inactive (0.63)	Active (0.52)	Active (0.98)	Inactive (0.71)	Inactive (0.62)
C3	High	Yes	91.7	No	No	No	Yes	No	Yes	1.632	0.099	600 mg/kg	Inactive (0.68)	Active (0.64)	Active (0.86)	Inactive (0.53)	Inactive (0.69)
C4	High	Yes	96.5	No	No	No	Yes	Yes	Yes	11.636	0.78	3000 mg/kg	Inactive (0.79)	Active (0.53)	Active (0.99)	Inactive (0.71)	Inactive (0.68)
C5	High	Yes	85.7	No	No	No	Yes	No	Yes	1.921	0.072	832 mg/kg	Inactive (0.74)	Active (0.61)	Active (0.96)	Inactive (0.54)	Inactive (0.70)
C6	High	Yes	99.6	No	No	No	No	No	No	1.667	0.14	4738 mg/kg	Inactive (0.76)	Inactive (0.52)	Active (0.95)	Active (0.51)	Inactive (0.93)
C7	High	No	93.0	No	Yes	No	No	Yes	No	1.243	0.47	221 mg/kg	Inactive (0.75)	Active (0.51)	Inactive (0.68)	Active (0.93)	Inactive (0.78)
C8	High	No	59.5	No	No	No	No	No	No	2.531	0.369	1100 mg/kg	Inactive (0.73)	Inactive (0.66)	Inactive (0.65)	Inactive (0.71)	Inactive (0.64)
C9	High	No	86.1	No	Yes	No	Yes	No	Yes	0.983	0.469	1600 mg/kg	Inactive (0.82)	Active (0.53)	Active (0.93)	Inactive (0.50)	Inactive (0.75)
C10	High	No	81.5	No	No	Yes	Yes	No	Yes	1.339	0.1	832 mg/kg	Inactive (0.74)	Active (0.62)	Active (0.89)	Inactive (0.58)	Inactive (0.71)
NL	High	No	97.6	No	Yes	Yes	Yes	Yes	No	1.259	0.703	729 mg/kg	Active (0.66)	Inactive (0.56)	Inactive (0.80)	Inactive (0.66)	Inactive (0.63)

### Molecular Docking and interactions analysis

Molecular docking of ligand and protein is widely acceptable to illustrate the inhibitory potential of several small organic molecules and it can reduce the efforts and time of wet lab study^124,125^. In this study the 3D conformers (41,581 in number) of 5820 filtered metabolites were downloaded from PubChem database and docked with the target protein (protein kinase CK2α subunit) to analyze the binding energy (ΔG) and binding affinity (Ki). There are several PDB files for the target protein in the database, but we have chosen PDB I’d: 3PE1 for our study with target protein due to its high resolution and it was available with its native ligand (CX-4945) bound to the catalytic (ATP) active site^29,31,126,127^.

**Figure 6:**
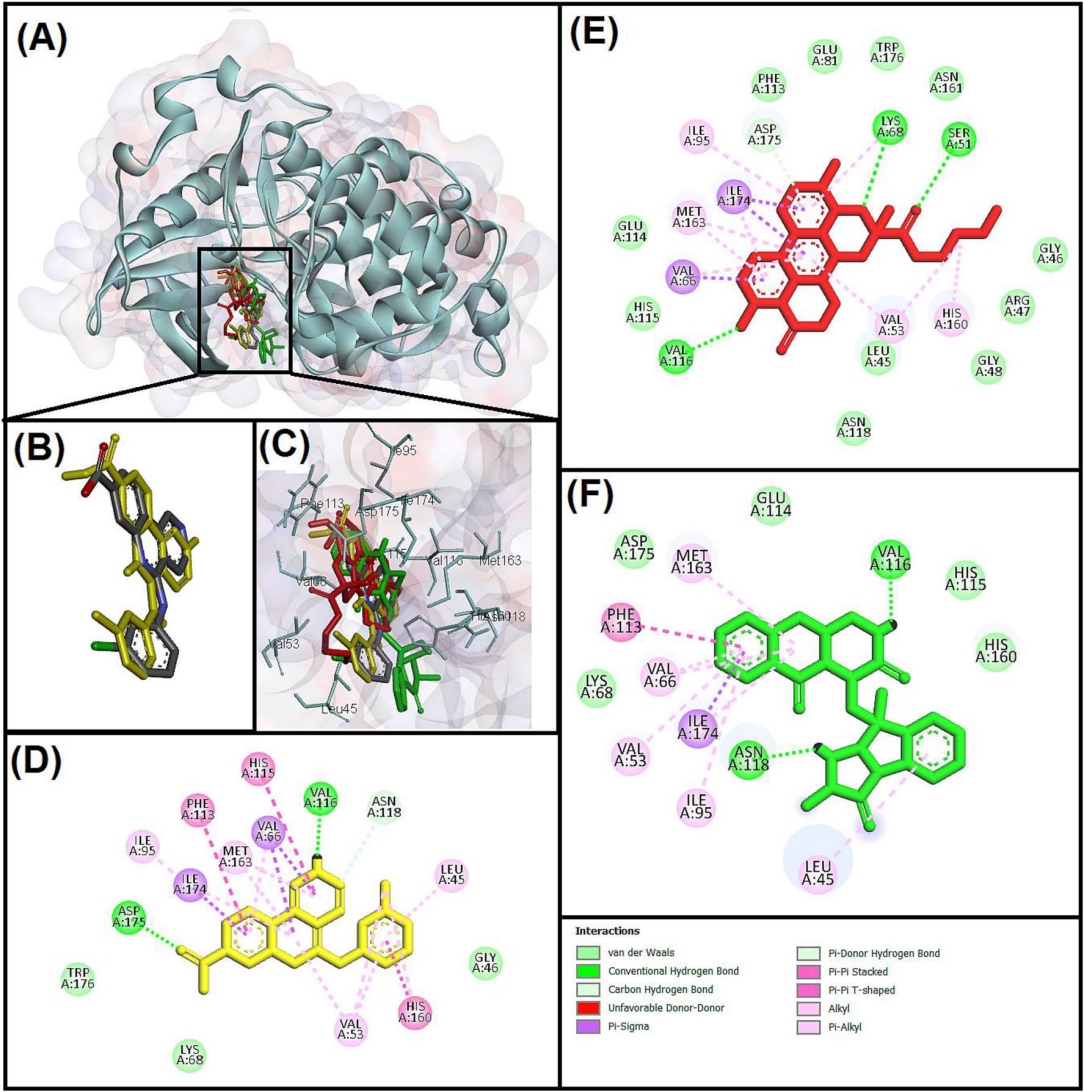
Molecular interactions analysis of best docked ligands with human protein kinase CK2α-subunit (3PE1) enzyme. (**A**) Superimpose image of docked ligands; (**B**) superimpose image of redocked (Yellow) and native (Grey) ligand; (**C**) zoom in image of all the docked ligands and interacting residues; two-dimensional structure and interacting residues of (**D**) CX-4945 (redocked), (**E**) Butyl Xanalterate (Red), (**F**) Fumiquinazoline Q (Green).

To validate the protocol the native ligand was redocked on its position and found that the ligand bound to almost similar residues and RMSD value (1.12) was in acceptable range (Fig. 6). Thereafter, all the fungal metabolites were docked individually on the active site (ATP binding site) of target protein after removal of native ligand (competitive inhibitor)^31,78^.

The results illustrated that only 35 fungal metabolites out of 5820 exhibited lower binding energy (ΔG: − 10.9 to − 11.7 kcal/mol) and better binding affinity (Kd: 9.77 × 10^7^ M^−1^ to 3.77 × 10^8^ M^−1^) than the native ligand (ΔG: − 10.8, Kd: 8.3 × 10^7^ M^−1^). The interaction of redocked complex of CX-4945 (native ligand) with target protein (3PE1) depicted through discovery studio visualizer revealed that the residues participated to stabilize the CX-4945 in the ac tive site of Protein kinase CK2α with three hydrogen bonds (VAL116, ASN118, ASP175), seventeen hydrophobic interactions (LEU45, VAL53, VAL66, ILE95, PHE113, HIS115, HIS160, MET163, ILE174), and two other interactions (LYS68, VAL116) (Table 4, Fig. 6A–D). Our results are in correspondence with previous studies where it was reported that the residues of Protein kinase CK2α subunit such as LYS68, and VAL116 formed hydrogen bond whereas VAL66, HIS160, and MET163 via hydrophobic bond interacted with CX-4945^31,128^. The binding energy (ΔG) of top 10 best hits (C1: − 11.7, C2: − 11.6, C3: − 11.5, C4: − 11.5, C5: − 11.4, C6: − 11.4, C7: − 11.4, C8: − 11.3, and C9: − 11.2, C10: − 11.2 kcal/mol) and binding affinity (C1: 3.77 × 10^8^, C2: 3.18 × 10^8^, C3: 2.69 × 10^8^, C4: 2.69 × 10^8^, C5: 2.27 × 10^8^, C6: 2.27 × 10^8^, C7: 2.27 × 10^8^, C8: 1.92 × 10^8^, C9: 1.62 × 10^8^, C10: 1.62 × 10^8^ M^−1^) exhibited their better potential to inhibit the target protein (3PE1) than all other fungus metabolites.

**Table 4:** Molecular interactions analysis and 2-D structure of best docked fungal metabolites with 3PE1.

Compound name	2-D structure	Hydrogen bond (Distance Å)	Hydrophobic (Distance Å)
CX-4945 (Silmitasertib) PubChem CID: 24748573 MF: C_19_H_12_ClN_3_O_2_ MW: 349.8 g/mol Redocked		VAL116 (2.29), ASN118 (3.68), ASP175 (2.03)	LEU45 (5.4), VAL53 (4.27, 4.57, 5.04), VAL66 (3.69, 3.94, 4.60), ILE95 (5.28), PHE113 (5.05), HIS115 (5.54), HIS160 (4.57, 5.15), MET163 (4.70, 4.87), ILE174 (3.82, 3.99, 5.08)
Butyl Xanalterate PubChem CID: 139590691 MF: C_24_H_22_O_7_ MW: 422.4 g/mol		SER51 (2.88), LYS68 (2.77), VAL116 (2.22), ASP175 (3.16)	VAL53 (4.46, 4.99), VAL66 (3.8, 4.58), LYS68 (5.49), ILE95 (5.14), HIS160 (5.42), MET163 (4.65, 5.27), ILE174 (3.64, 3.87, 5.07)
Fumiquinazoline Q PubChem CID: 139584842 MF: C_23_H_21_N_5_O_4_ MW: 431.4 g/mol		VAL116 (2.61), ASN118 (1.95),	LEU45 (4.67), VAL53 (5.36), VAL66 (4.23, 4.98), ILE95 (5.44), PHE113 (4.53), MET163 (4.7), ILE174 (3.94, 4.34)

All the best hits of fungus metabolites interacted with the same catalytic active site pocket of CK2α subunit where CX-4945 gets bind (Fig. 6A–C). Based on better binding energy, binding affinity, acceptable range of ADMET properties, high LD_50_ dose, and least toxicity (non-carcinogenic and non-mutagenic) we have finalized two fungal metabolites (PubChem compound I’d: 139590691 and 139584842) suitable for drug candidate hence we performed their molecular interaction analysis (Table 4).

Our molecular interaction results (Table 4) illustrated that Butyl Xanalterate and 3PE1 complex was stabilized by several interactions mentioned as Donor–Acceptor among them four hydrogen bonds was formed between SER51:HG—Ligand:O, LYS68:HZ2—Ligand:O, VAL116:HN—Ligand:O, and ASP175:HN—Ligand. Moreover, twelve hydrophobic interactions were formed to stabilize the complex in which three Pi-sigma (VAL66:CG2—Ligand, ILE174:CD—Ligand, and ILE174:CD—Ligand), one alkyl (VAL53—Ligand), and eight Pi-alkyl (HIS160—Ligand, Ligand—VAL53, Ligand—VAL66, Ligand—MET163, Ligand—MET163, Ligand—ILE174, Ligand—LYS68, and Ligand—ILE95) interactions were noticed (Table 3, Fig. 6E). Eleven residues were also involved in stabilizing the complex via Van Der Waals interaction (LEU45, GLY46, ARG47, GLY48, GLU81, PHE113, GLU114, HIS115, ASN118, ASN161, and TRP176) (Fig. 6E). The Fumiquinazoline Q and target protein (3PE1) were stabilized by two hydrogen bonds between Ligand:H—VAL116:O, and Ligand:H—ASN118:OD1. Whereas nine hydrophobic interactions were also observed among them one Pi-sigma between ILE174:CD—Ligand, one Pi-Pi Stacked between PHE113—Ligand, and seven Pi-alkyl between Ligand—VAL66, Ligand—MET163, Ligand—ILE174, Ligand—VAL53, Ligand—VAL66, Ligand—ILE95, and Ligand—LEU45, respectively (Table 3, Fig. 6F). Moreover, five residues of active site were involved in Van Der Waals interactions (LYS68, GLU114, HIS115, HIS160, ASP175) between ligand and target protein (Fig. 6F).

The molecular docking also predicted the binding score of interacted molecules which revealed that the binding energy and binding affinity of Butyl Xanalterate (− 11.7 kcal/mol and 3.77 × 10^8^ M^−1^) is better than all other fungal metabolites including by Fumiquinazoline Q (− 11.3 kcal/mol and 1.92 × 10^8^ M^−1^). These both fungal metabolites are 4.57 and 2.33 folds, respectively better active than reference standard (CX-4945). Our results are in correspondence with previous studies where Oramas-Royo et al.^30^ docked some synthesized compounds with Protein kinase CK2 and reported that the compounds fully occupied the active site in adenine region (VAL53, VAL66, VAL116, and MET163), hydrophobic region I (PHE113, ILE95, and ILE174), and hydrophobic region II (LEU45, and HIS115).

### Molecular dynamic (MD) simulation

The docked ligand–protein complexes were subsequently investigated using molecular dynamics simulations. MD simulations of 100 ns were performed for three complex systems: Silmitasertib (control) and two identified compounds, Butyl Xanalterate and Fumiquinazoline Q to assess the dynamic behavior and stability of the complexes, particularly under physiological conditions. The MDS trajectories have been used to calculate the root-mean square deviation (RMSD), root-mean square fluctuation (RMSF), ligand–protein interactions analyses and Radius of gyration (rGyr) of the complexes in order to study their structural stabilities, binding modes, and conformational flexibilities.

### Root-mean-square deviation

**Figure 7:**
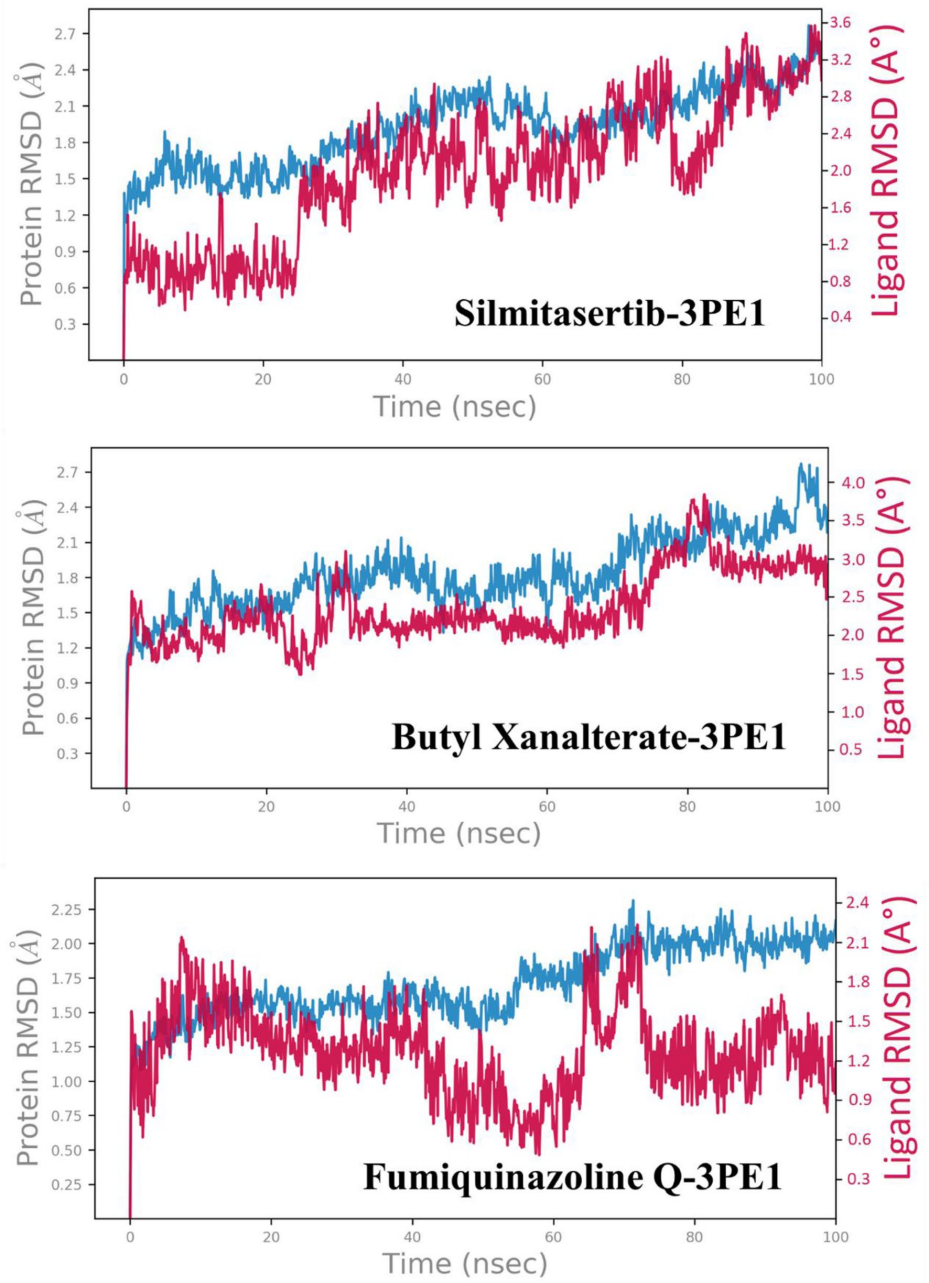
Protein and ligand RMSD for the trajectory of each system. Protein RMSD is shown in grey while Ligand RMSD is shown in red.

The RMSD is a measure of the mean deviation in atom displacement in the protein–ligand complex during a certain time frame in comparison to the initial frame. To evaluate the complex's stability over time, the computation is performed for each frame in the simulation trajectory. During MD simulation, a lower RMSD value shows a more stable protein–ligand complex, whereas a higher RMSD value denotes a less stable complex^129,130^. According to the Cα backbone reference frame, the RMSD of human protein kinase CK2α subunit (3PE1) during simulation is shown in Fig. 7. The RMSD of the protein Cα atoms rise substantially for an initial 1500 ps then stabilized between 1.8 and 2.7 Å for the rest of the simulation. Ligand RMSD for Silmitasertib fitted on the 3PE1 backbone showed small deviations for the initial 23 ns, then after slight fluctuation, RMSD remained in the range of 1.6–3.6 Å for the remaining simulation. The maximum RMSD value of the Cα backbone of the Butyl Xanalterate-3PE1complex is 2.7 Å, indicating that the complex was retained continuously throughout the simulation time. The Ligand RMSD for Butyl Xanalterate increased initially due to equilibration and then showed RMSD over a range of 2.0–3.5 Å throughout the simulation, which is comparable to that reported for the Silmitasertib-3PE1 system and found within the recommended cutoff of 3 Å. In the Fumiquinazoline Q-3PE1 complex, the RMSD of the protein’s backbone was fluctuating between 1.25 and 2.25 Å with an average of 1.80 Å. The RMSD of the Fumiquinazoline Q corresponding to the backbone of the protein was found to fluctuate between 0.6 and 2.1 Å and mean RMSD was found to be below 1.2 Å throughout the simulation time. In this complex, the highest fluctuation was observed at 69–77 ns, which is up to 2.1 Å. It could perhaps be noted that none of the RMSD fluctuations exceeded the allowable limit of 3 Å.

### Root-mean-square fluctuation

**Figure 8:**
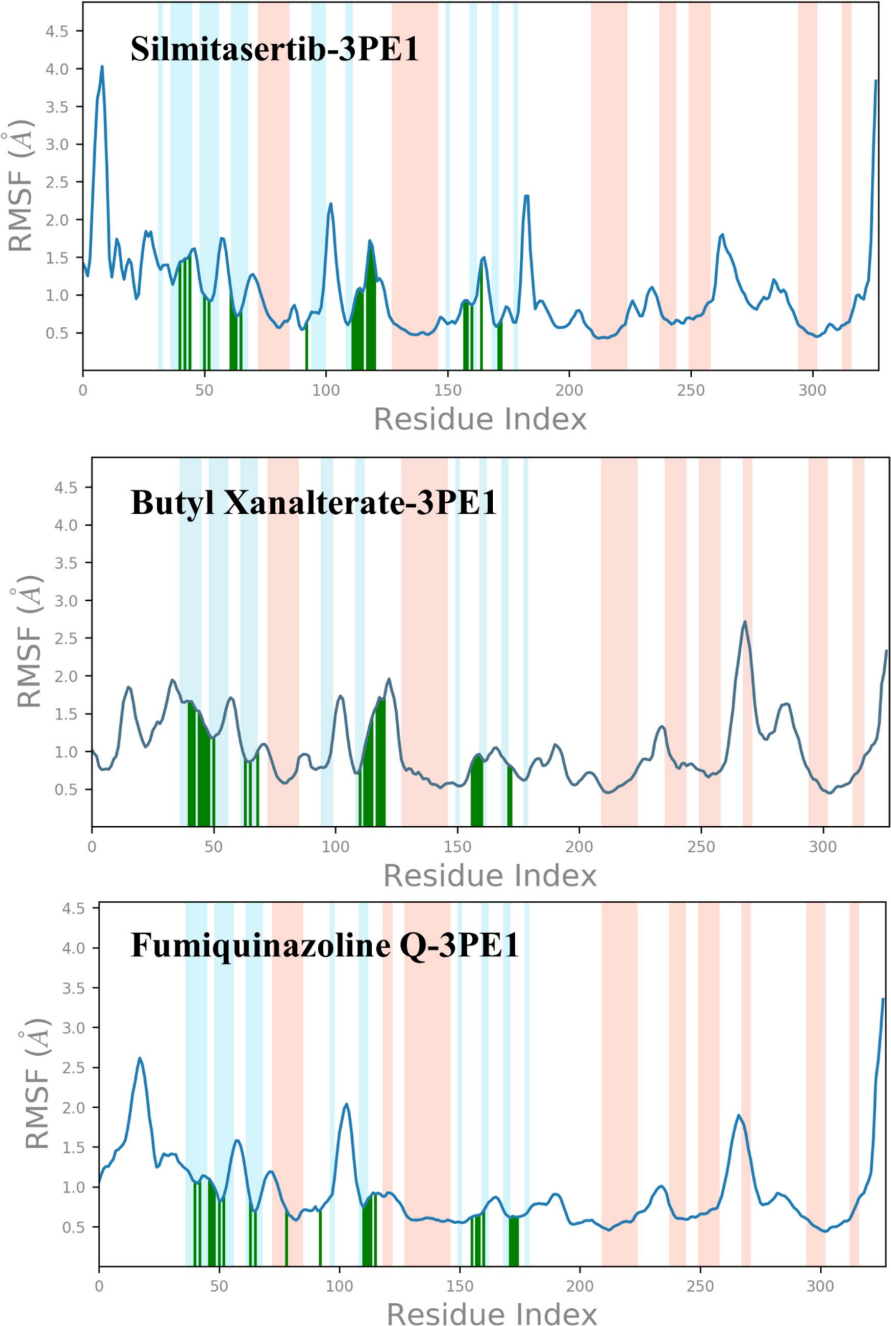
RMSF of human protein kinase CK2α subunit upon ligand binding, amino acid that interacted with ligand marked in green vertical bar.

Further, the flexibility and mobility of the human protein kinase CK2α subunit protein amino acid residues were assessed, in contrast to a reference structure. A large RMSF value indicates flexibility, loose bonding, or the presence of loops in the protein structure; a low RMSF value indicates stability as well as the prevalence of secondary structural elements such as sheets and helices^131,132^. In RMSF plot, the α-helical and β-strand portions are shown in red and blue color shades, respectively, while the loop area is shown in white. The RMSF values varied from 0.8 to 4.0 Å, 0.7 to 2.5 Å and 0.6 to 3.1 Å for the Silmitasertib-3PE1, Butyl Xanalterate-3PE1, and Fumiquinazoline Q-3PE1complexes, respectively. The residues in the range of 43–68, 114–123, and 160–172 that interact with the ligands, which are highlighted in green-colored vertical bars, have less than 2 Å RMSF values (Fig. 8). The fluctuations are most noticeable in the protein's tails (N- and C-termini) and unstructured sections (loop regions). The insignificant fluctuation of the active site and main chain atoms revealed that the conformational changes were modest, confirming that the reported lead compound was snugly bound within the cavity of the human protein kinase CK2α subunit protein binding pocket. Overall, the whole protein structure emerges in stable fluctuations in three complexes.

### Radius of gyration

**Figure 9:**
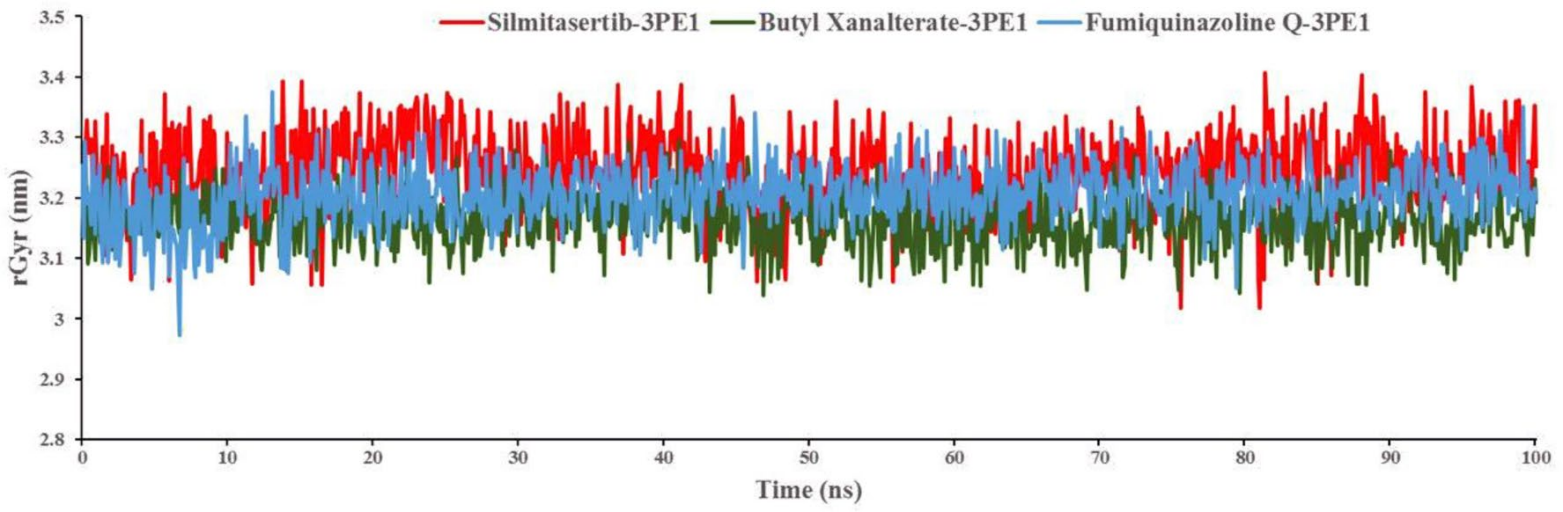
The radius of gyration of human protein kinase CK2α subunit bound with the identified ligands.

The folding changes and rigidity of the human protein kinase CK2α subunit associated with all identified small molecules were investigated using rGyr, which was computed using MD simulation trajectories. The rGyr of the human protein kinase CK2α subunit protein bound with Silmitasertib, Butyl Xanalterate, and Fumiquinazoline Q were plotted against the time of simulation, as shown in Fig. 9. The rGyr of all the systems was observed to be between 3.1 and 3.4 nm. There was no significant divergence found in any of the systems. During the MD simulation study, the human protein kinase CK2α subunit bound to the identified molecules demonstrated a consistent equilibrated fluctuation in rGyr. The difference between the highest and least rGyr measurements of each system may be used to calculate the range of variation. The difference in rGyr values of kinase CK2α subunits bound with Silmitasertib, Butyl Xanalterate, and Fumiquinazoline Q was revealed to be 0.389, 0.256, and 0.403 nm, respectively. The rGyr data acquired can undoubtedly represent the rigidity and compactness of the human kinase CK2α subunit protein structure during the MD simulation for all compounds bound state.

### Analysis of Protein ligand interaction

**Figure 10:**
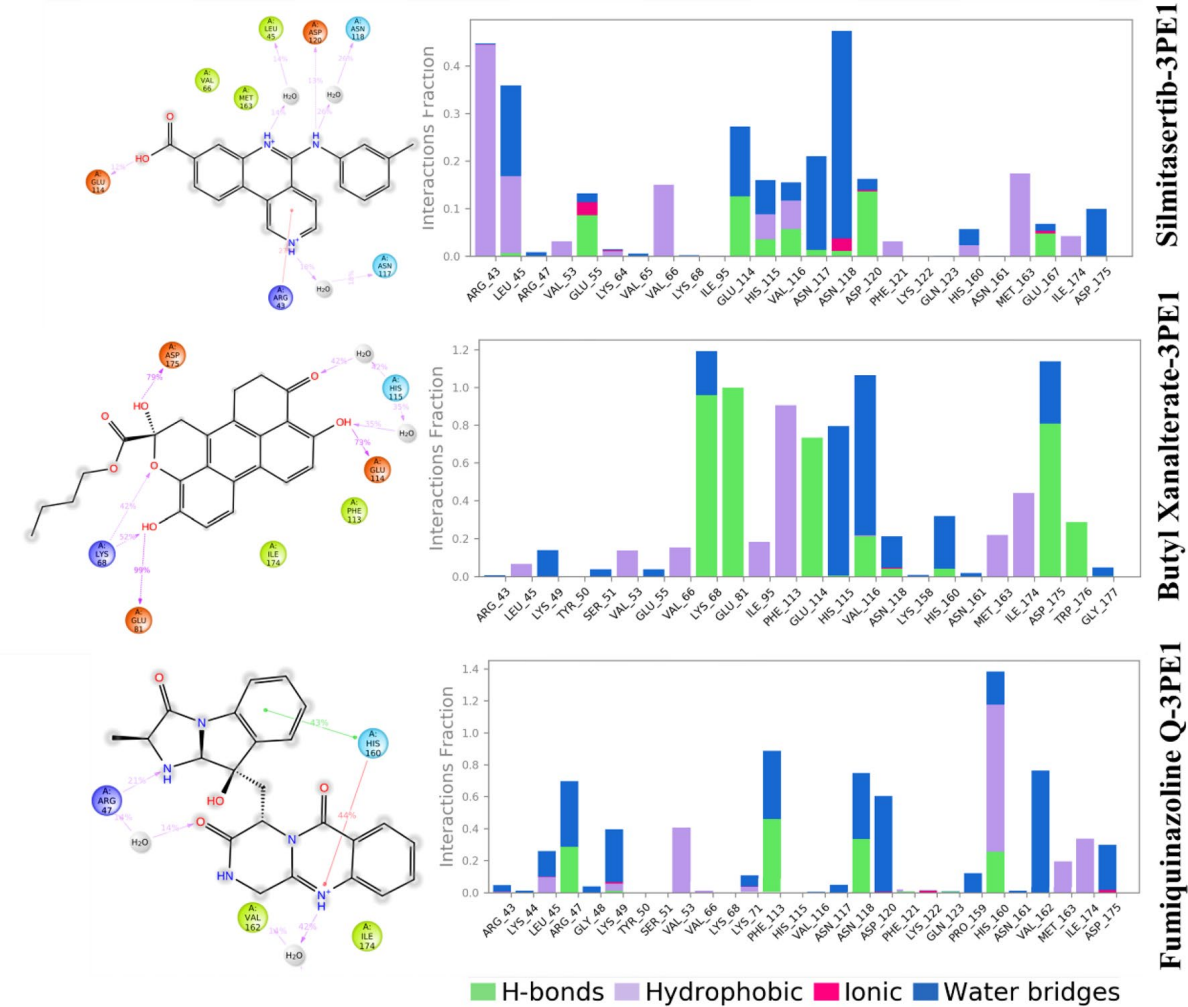
Histogram shows protein residues that interact with the ligand over the course of the trajectory.

Throughout the simulation, protein interactions with the ligand were monitored. These interactions can be categorized, examined. Protein–ligand interactions were classified into four types: hydrogen bonds, ionic interactions, hydrophobic interactions, and water bridges. The histogram in Fig. 10, depicts the types of interactions of Silmitasertib, Butyl Xanalterate, and Fumiquinazoline Q with the human protein kinase CK2α subunit during the simulation. Silmitasertib forms strong hydrogen bonds with Glu55, Glu114, His115, Val116, Asp120, and Glu167 and some ionic or polar interactions between two oppositely charged atoms with Glu55 and Asn118. Residues Leu45, Glu114, Asn118, and Asn117 interacted with Silmitasertib through water-mediated hydrogen bonding at 14%, 13%, 24%, and 16%, respectively. In the case of the Butyl Xanalterate-3PE1 complex, 3PE1protein residues Lys68, Glu81, Glu114, and Asp175 exhibited more than 65% hydrogen bond interactions. In this complex, a significant hydrophobic interaction with residue Phe113 was observed, accounting for 80% of the MD simulation trajectory. Protein ligand contact mapping of the complex protein Fumiquinazoline Q-3PE1 protein reveals that the residues Arg47, Phe113, Asn118, and His160 had more than 20% hydrogen bond interactions with the lead compound Fumiquinazoline Q. Fumiquinazoline Q primarily interacts with Arg47, Lys49, Phe113, Asn118, Asp120, Val 162, and Asp175 through water bridges for more than of 14% of the simulation, respectively. Overall, the simulation showed that the reported compounds were stabilized by hydrogen bonding, amino-acid driven water bridges, and hydrophobic interaction.

### Post simulation binding free energy

MM/GBSA is a common and rigorous approach for post-simulation binding free energy prediction because it considers protein flexibility, entropy, and polarizability, which are often overlooked in docking protocols. The binding energy estimation approach based on molecular mechanics-generalized Born surface area (MM/GBSA) allows for the identification of ligands that bind efficiently with receptors One of the most significant goals in bimolecular investigations is to calculate the free energy of binding precisely because it is responsible for driving all molecular activities such as chemical reaction, molecular recognition, association, and protein folding^133^. Hence validity of compounds identified by docking and MD simulations was investigated further by using MMGBSA binding free energy estimate calculations. The post-simulation MM/GBSA was estimated at every 10th frame from frame 0–1001, totaling 100 conformations (every ns) of each simulated complex, and the average binding energies with standard deviation are given in Table 5. MM/GBSA binding energy statistics show that the cumulative contributions of Coulombic, H-bond, Lipo, and vdW interactions have a significant impact on ΔG_Bind_.

**Table 5:** Post simulation Components of binding free energy for protein–ligand complexes estimated using MM-GBSA analysis.

Complex name	MMGBSA (kcal/mol)					
	ΔG_Bind_	ΔG_Coulomb_	ΔG _H_bond_	ΔG_Lipo_	ΔG_Solv_GB_	ΔG_vdW_
Butyl Xanalterate-3PE1	− 74.41	− 27.65	− 3.66	− 21.59	27.58	− 51.47
Fumiquinazoline Q-3PE1	− 34.6	42.53	− 0.73	− 8.76	− 37.66	− 30.96
Silmitasertib-3PE1	− 40.86	49.61	− 0.63	− 10.68	− 45.53	− 31.99

The calculated average ΔG_Bind_ of the complex Butyl Xanalterate, Fumiquinazoline Q and Silmitasertib, in complex with the protein kinase CK2α subunit was found − 74.41 kcal/mol, − 34.6 kcal/mol and − 40.86 kcal/mol, respectively. A more negative value shows stronger binding, the highest ΔG_Bind_ was seen for the Butyl Xanalterate-3PE1complex, this value is significantly higher than that observed for the standard Silmitasertib-3PE1 complex. Furthermore, vdW and H-bond interactions are important contributors to ligand binding in all cases; however, it seems ΔG_Lipo_ may also significantly affect the binding free energy of Butyl Xanalterate-3PE1 complex. Thus, based on binding free energy values, the order of best compounds is Butyl Xanalterate > Silmitasertib > Fumiquinazoline Q.

The present study should be viewed with some considerations. First, the investigations that were conducted to show the association of *CSNK2A1* with i) short TTFT, ii) short OS, and iii) survival and proliferation-dependent pathways in CLL cells was entirely based on gene expression rather than protein expression (which is the main functional molecule in a cell). While the central dogma of molecular biology states that gene expression correlates with protein expression, some genes do not follow this pattern^134^. In this work, we carefully searched for CLL proteomics data sets with available clinical information about OS and TTFT in public depositories, such as National Center for Biotechnology Information (NCBI), Protein Atlas and The Cancer Genomic Atlas (TCGA), but we could not find any. The only available data sets that fit with the goal of our study were the two CLL transcriptomics data sets used here. Therefore, in our future work, we will evaluate the protein expression and the kinase activity of CK2α in CLL cells isolated from CLL patients to confirm its prognostic value in the disease. Second, although the identification of Butyl Xanalterate as a competent inhibitor of CK2α was based on rigorous in silico approach, confirming this finding using wet lab experiments is needed. Consequently, in our future work, we will compare the therapeutic potential of Butyl Xanalterate with that of CX-4945 using wet lab experimental settings to determine if Butyl Xanalterate is stronger inhibitor of CK2α and more competent than CX-4945 in causing CLL cells undergo apoptosis.

## Conclusion

Overall, this study built a rational for targeting CK2α for CLL therapy by reporting i) an association between the expression of *CSNK2A1* with short TTFT and short OS, ii) a correlation the expression of genes implicated in CLL survival and proliferation-dependent pathways with the expression of *CSNK2A* in CLL cells, iii) identification of CK2α interaction patterns that associate with the aggressiveness of CLL. Next, utilizing different computational tools identified Butyl Xanalterate (from 19,967 fungal metabolite) to exhibit a strong inhibitory potential against CK2α (*CSNK2A1*), and this inhibition was found to be even better than the approved inhibitor (CX-4945). Butyl Xanalterate was found to be highly safe as it did not show any toxicity, do not cross BBB, has “good” in metabolism, and has high oral bioavailability. The MD simulations of 100 ns and MM-GBSA study concluded that Butyl Xanalterate has stable and strong interactions with human kinase CK2α. Taken together, these findings proposed Butyl Xanalterate as a competent inhibitor of CK2α that could be employed for CLL therapy. Our future work will apply wet lab experimental settings to assess the potential of Butyl Xanalterate to target CK2α and induce apoptosis in CLL isolated from CLL patients, especially those with progressive and aggressive form of the disease. In this scenario, the therapeutic potential of Butyl Xanalterate will be compared with CX-4945 to determine if Butyl Xanalterate is more competent compared with CX-4945 as our currently study delineated.

## Supplementary Information

Supplementary Information.

## Data Availability

All datasets generated for this study are included in the manuscript.
